# A High-Throughput Screening System Based on Fluorescence-Activated Cell Sorting for the Directed Evolution of Chitinase A

**DOI:** 10.3390/ijms22063041

**Published:** 2021-03-16

**Authors:** Gheorghita Menghiu, Vasile Ostafe, Radivoje Prodanović, Rainer Fischer, Raluca Ostafe

**Affiliations:** 1 Institute for Biology VII, Molecular Biotechnology, RWTH Aachen University, Worringerweg 1, 52074 Aachen, Germany; gheorghita.menghiu@e-uvt.ro (G.M.); fische70@purdue.edu (R.F.); 2 Advanced Environmental Research Laboratories, Department of Biology–Chemistry, West University of Timisoara, Oituz 4, 300086 Timisoara, Romania; vasile.ostafe@e-uvt.ro; 3 Faculty of Chemistry, University of Belgrade, Studentski trg 12-16, 11000 Belgrade, Serbia; rprodano@chem.bg.ac.rs; 4 Departments of Biological Sciences and Chemistry, Purdue University, 207 S. Martin Jischke Dr., West Lafayette, IN 47907, USA; 5 Purdue Institute of Inflammation, Immunology and Infectious Disease, Molecular Evolution, Protein Engineering and Production, Purdue University, 207 S. Martin Jischke Dr., West Lafayette, IN 47907, USA

**Keywords:** FACS, protein engineering, error-prone PCR, mutants, bactericidal effect, improved enzymes, fluorescence assay

## Abstract

Chitinases catalyze the degradation of chitin, a polymer of *N*-acetylglucosamine found in crustacean shells, insect cuticles, and fungal cell walls. There is great interest in the development of improved chitinases to address the environmental burden of chitin waste from the food processing industry as well as the potential medical, agricultural, and industrial uses of partially deacetylated chitin (chitosan) and its products (chito-oligosaccharides). The depolymerization of chitin can be achieved using chemical and physical treatments, but an enzymatic process would be more environmentally friendly and more sustainable. However, chitinases are slow-acting enzymes, limiting their biotechnological exploitation, although this can be overcome by molecular evolution approaches to enhance the features required for specific applications. The two main goals of this study were the development of a high-throughput screening system for chitinase activity (which could be extrapolated to other hydrolytic enzymes), and the deployment of this new method to select improved chitinase variants. We therefore cloned and expressed the *Bacillus licheniformis* DSM8785 chitinase A (*chiA*) gene in *Escherichia coli* BL21 (DE3) cells and generated a mutant library by error-prone PCR. We then developed a screening method based on fluorescence-activated cell sorting (FACS) using the model substrate 4-methylumbelliferyl β-d-*N*,*N*′,*N*″-triacetyl chitotrioside to identify improved enzymes. We prevented cross-talk between emulsion compartments caused by the hydrophobicity of 4-methylumbelliferone, the fluorescent product of the enzymatic reaction, by incorporating cyclodextrins into the aqueous phases. We also addressed the toxicity of long-term *chiA* expression in *E. coli* by limiting the reaction time. We identified 12 mutants containing 2–8 mutations per gene resulting in up to twofold higher activity than wild-type ChiA.

## 1. Introduction

Chitin is an insoluble, high-molecular-weight polymer comprising linear chains of β(1,4)-linked *N*-acetyl-d-glucosamine (Figure S1). It is the second most abundant biopolymer on the earth after cellulose, and is a major structural polysaccharide in insects, crustaceans, and fungi [1]. Massive quantities of chitin waste are generated by the processing of crabs, shrimps, and lobsters for food [2]. The natural degradation of chitin takes a long time because the polymer is insoluble and highly crystalline, and the accumulation of chitin waste is increasingly seen as an environmental burden, especially in countries with a prominent shellfish industry [3,4]. Efficient and environmentally friendly methods are required to convert chitin into valuable products such as chitosan oligomers, which are shorter-chain soluble products containing mixtures of β-(1,4)-linked d-glucosamine and *N*-acetyl-d-glucosamine. They are known for their bioactive properties, including antimicrobial, immunomodulatory, and antioxidant activities that can be exploited in medicine, agriculture, food processing, water purification, and cosmetics [5,6,7]. The enzymatic production of chitosan oligomers from chitin is favored over physical and chemical treatments that involve harsh conditions. Enzymes that catalyze the hydrolysis of chitin are known as chitinases (EC 3.2.1.14), but the practical application of natural chitinases is limited by their low activity [8].

The disadvantages of wild-type chitinases can be overcome by combining heterologous expression with the redesign of chitinases using molecular evolution, which involves consecutive rounds of mutation and selection to isolate clones with improved properties. Directed evolution has been applied to a variety of enzymes with industrial applications, such as glucose oxidase [9,10], P450 monooxygenases [11], cellulases [12], peroxidases [13], cellobiose dehydrogenase [14], transaminases [15], esterases [16], lipases [17], proteases [18], and many more. However, there are only a few examples of the directed evolution of chitinases [19,20,21].

Several methods are readily available for the introduction of mutations at the gene level and have been used reliably for years [22]. Random mutagenesis by error-prone PCR is one such method that is widely used to generate enzyme libraries of up to 10^8^–10^12^ variants [23,24]. However, complete screening of such complex libraries is not possible using any current method. The traditionally slow process of library screening has been accelerated in recent years by high-throughput screening using integrated robotic systems that transport assay microtiter plates (MTPs) from station to station for sample and reagent addition, mixing, incubation, and detection. This allows the sampling of 10^4^–10^6^ variants in months [25,26,27]. But screening can be very expensive in terms of upfront investment in equipment as well as running costs associated with plastic consumables and the substrates needed for the reactions.

In the last decade, ultrahigh-throughput screening systems based on flow cytometry have been used for the screening of various enzymes [9,28,29,30,31,32,33]. Fluorescence-activated cell sorting (FACS) has gradually emerged as a tool for the screening of enzyme libraries due to its high sensitivity and ability to test up to 10^7^ enzyme variants per day [34], allowing the isolation of variants with improved activity, altered substrate specificity, or even novel functions [35]. Despite its great potential for enzyme evolution, FACS screening is still not widely used due to limited assay compatibility and the need to preserve the genotype–phenotype linkage during screening. Robotic or manual screening in MTPs is still the method of choice in most directed evolution laboratories due to the compatibility of the system with a variety of common assay formats such as luminescence, absorbance, fluorescence, and even mass spectrometry. Another advantage is that the link between the phenotype and mutant genotype is preserved by enclosing each reaction in a single well during the screening process.

In FACS-based screening, the detection assay is limited to fluorescence and the connection between genotype and phenotype is more difficult to maintain. One solution is to create artificial compartments around the cells that would keep the product of the enzymatic reaction (phenotype) connected to the cells expressing the mutant enzyme (genotype). One way to create such a barrier is the use of water-in-oil-in-water double emulsions. This in turn limits the pool of usable substrates because hydrophobic compounds diffuse out of the emulsion, and many fluorescent products are hydrophobic. The only commercially available substrate for chitinase is β-*N*,*N*′,*N*″-triacetylchitotrioside (4MUTC), which forms a hydrophobic product, so we considered this as a model to overcome the diffusion of a hydrophobic product from the emulsion compartments and therefore increase the range of substrates that can be used with FACS-based screening for other enzyme classes.

Here we describe the development of a FACS-based screening assay to identify improved variants of the chitinase ChiA from *Bacillus licheniformis* DSM8785. This enzyme was chosen as a starting point for our experiments because the sequence has been successfully expressed in *Escherichia coli* [4]. The *chiA* gene was cloned in the periplasmic expression vector pET22b(+) and expressed in *E. coli* BL21 (DE3) cells for molecular evolution by error-prone PCR, aiming to generate variants with greater activity. The periplasmic space was targeted because enzyme activity can be detected without cell lysis, depending on the substrate size. We optimized several parameters such as the *chiA* expression time and the duration of enzyme incubation with the fluorogenic substrate 4MUTC in the emulsion, and tried various additives in the emulsion system in order to keep the hydrophobic product from diffusing out of the droplet. The selected ChiA mutants were tested in MTP assays to compare their activities to the wild-type variant.

## 2. Results and Discussion

### 2.1. Development of the FACS Assay

Only three previous studies describe the directed evolution of chitinases, all of which involve low-throughput screening methods based on agar plate or MTP assays [19,20,21]. The natural substrate of ChiA is chitin or partially acetylated chitosan, because it has an absolute requirement for *N*-acetyl-d-glucosamine and cleaves glycosidic bonds at random internal sites immediately downstream of an *N*-acetyl-d-glucosamine unit [36]. Chitinase activity on chitin or chitosan is usually measured by detecting the reducing ends of polysaccharide fragments with 3,5-dinitrosalicylic acid (DNS) [37]. However, the DNS assay and other reducing-end detection methods are end-point reactions, so multiple aliquots from the reaction mix must be sampled at different time points. This method is therefore cumbersome and unsuitable for high-throughput screening. In addition, FACS requires the formation of a fluorescent product.

FACS-based screening methods using whole cells and double emulsions have been reported for multiple enzymes, but none of them have used hydrophobic products in the screening steps [9,28,29,30,31,32,33]. Hydrophobic products are challenging because they diffuse from the emulsion compartments, but 4MUTC is the only commercially available substrate for chitinase. The use of other substrates would require de novo organic synthesis, which is beyond the scope of our study. Furthermore, umbelliferyl substrates are commonly used for multiple enzymes (xylanase, glucose oxidase, glycosidase, cellulase, deacetylase, phosphatase, and others), providing additional justification for attempting to make FACS-based screening compatible with hydrophobic substrates [38,39,40]. We therefore selected the synthetic fluorogenic substrate 4MUTC for the detection of chitinase activity. 

**Figure 1:**
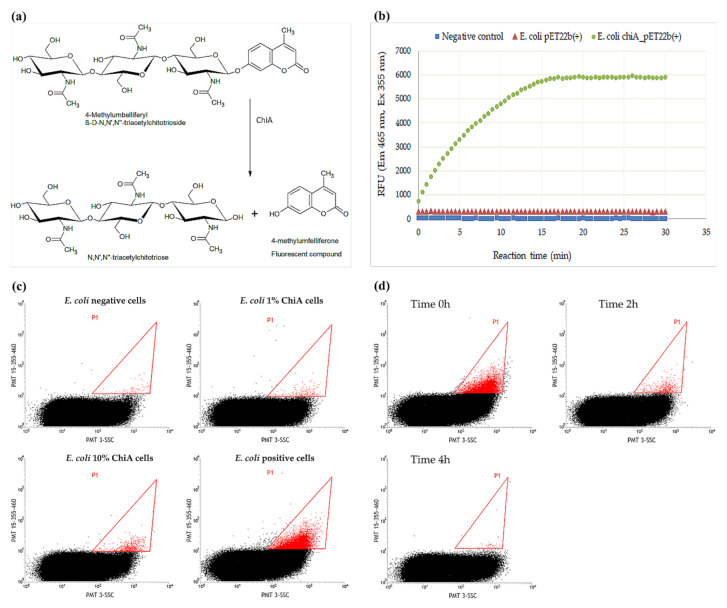
The synthetic fluorogenic substrate 4-methylumbelliferyl β-d-*N*,*N*′,*N*″-triacetyl chitotrioside (4MUTC) was used for the detection of chitinase activity. (**a**) ChiA cleaves 4MUTC to generate the fluorescent product 4-methylumbelliferone (4MU) and *N*,*N*′,*N*″-triacetylchitotriose. (**b**) MTP fluorescence assay using 4MUTC as the substrate for ChiA. (**c**) FACS response of *E. coli* cells containing ChiA in emulsions. Samples comprising 100% positive (*E. coli chi*A_pET22b+) or 100% negative cells (*E. coli* pET22b+) as well as samples comprising different proportions of positive and negative cells were tested. The positive droplet population is shown in red. (**d**) Decay of the FACS response of *E. coli* cells containing ChiA over time, in double emulsions.

Esters of 4-methylumbelliferone (4MU) do not fluoresce unless cleaved to release the fluorophore. The assay for ChiA activity is therefore based on the hydrolysis of 4MUTC and the detection of the cleavage product 4MU, which emits a signal at 460 nm when excited at 365 nm (Figure 1a). The assay was verified in MTP format, comparing *E. coli* cells expressing *chiA* to those transformed with the empty vector as negative controls (Figure 1b).

The assay was also tested in double emulsions, using samples comprising 100% positive or 100% negative cells as well as samples comprising different proportions of positive and negative cells, mimicking real libraries (Figure 1c). We found that the assay was sensitive to the proportion of positive cells in the mix because the positive droplet population (red) increased in line with the proportion of cells expressing *chiA* available in the mix, in agreement with earlier studies [9,41]. However, we observed a decline in the level of fluorescent product if the emulsions were incubated for longer time (Figure 1d), probably reflecting the hydrophobic nature of 4MU, which can diffuse out of the internal water phase of the emulsion system through the oil phase and, potentially, into other droplets.

If 4MU leaks from the emulsion droplet containing the positive cell in which it was created, this risks the generation of false-negative results. Furthermore, if the escaping 4MU enters droplets that lack chitinase activity, this risks the generation of false-positive results. The first issue can be addressed by inspecting the flow cytometry recordings over time but the second cannot, yet would nevertheless influence the sorting efficiency. To ensure that 4MU remains within the emulsions and does not travel across the hydrophobic layers, we added methyl-β-cyclodextrin (MCD) and 2-hydroxypropyl-β-cyclodextrin (HCD) to the droplet phases to test their retention ability (the structures of MCD and HCD are presented in Figure S2).

**Figure 2:**
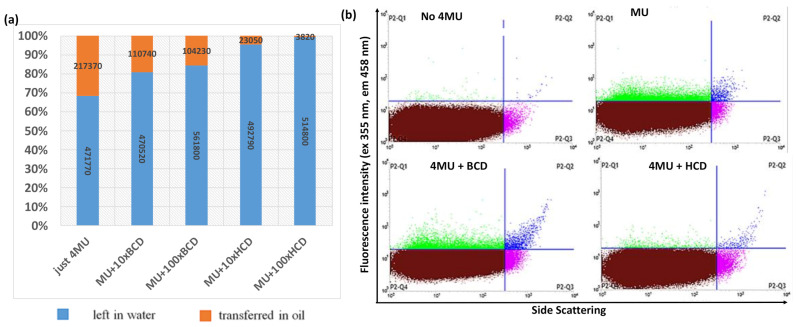
Analysis of cyclodextrins for the retention of 4-methylumbelliferone (4MU) in a simple biphasic mixture and single emulsions. (**a**) Partition coefficient of 4MU between the oil and water phases of a simple mixture, showing the relative fluorescent units (RFUs) measured in each phase. (**b**) FACS analysis of 4MU, 4MU+MCD, and 4MU+HCD in the external phase of single oil-in-water emulsions (ABIL EM-CMC-Triton X-100).

Cyclodextrins (CDs) are cyclic oligosaccharides with a hydrophilic outer surface and a lipophilic central cavity, allowing them to act as molecular containers by entrapping guest particles or complex inclusions [42]. CDs have therefore been used to prevent crosstalk between emulsion droplets [43]. The partition of 4MU between the water and oil phases used for the preparation of double emulsions was determined by measuring the fluorescence in a simple biphasic mixture after incubation with 4MU for 1 h. Different types and concentrations of CDs were added to the water phase to evaluate their ability to retain 4MU, revealing that a 100-fold molar excess of HCD over 4MU keeps more than 95% of 4MU in the water phase (Figure 2a).

To test the performance of HCD in a real emulsion system but with lower complexity than the double emulsions used for the encapsulation of cells, we created a single oil-in-water emulsion using the same oil/detergent combination. We then added 4MU to the external water phase before the emulsions were diluted. The samples were incubated at room temperature for 1 h and diluted 10-fold for FACS analysis. The presence of positive (green droplets) in the P2-Q1 quarter confirmed that 4MU entered the oil system in the absence of CDs but was retained more efficiently when HCD was present in the water phase (Figure 2b). We also tested a perfluorinated oil and detergent that should dissolve neither hydrophilic nor hydrophobic compounds [44], but 4MU was highly soluble in the oil and the addition of CDs did not prevent the uptake of 4MU by the oil phase (Figure S3).

**Figure 3:**
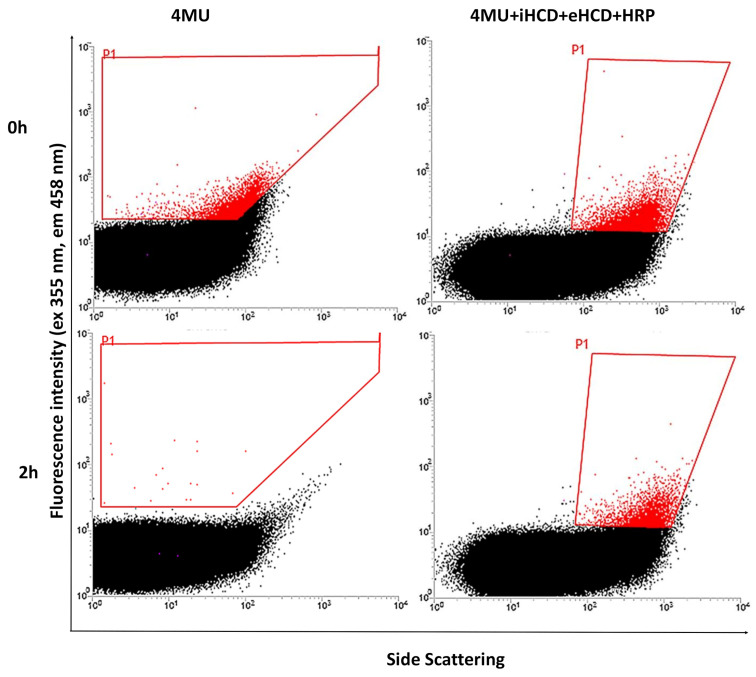
FACS analysis of 4-methylumbelliferone (4MU), 4MU+HCD in the internal phase (iHCD) and external phase (eHCD)+HRP, in double water-oil-water emulsions, after reaction for 2 h.

The single-emulsion experiments confirmed that CDs are necessary to limit the escape of 4MU from the water phase containing positive cells expressing ChiA, and we assumed that the same was likely to be the case for double emulsions. To limit the likelihood of cross-talk between droplets even further, we also added HCD to the external water phase of the double emulsions to provide insurance in case the HCD in the internal aqueous compartment was insufficient to prevent the leakage of 4MU or in the event of disruption of the oil layer, which would make the inner and outer water phases contiguous and would dilute the HCD in the inner phase. Using these optimized conditions, we prepared double emulsions with 4MU in the inner water phase, with and without HCD also in the external water phase. As a third variant, we also added horseradish peroxidase (HRP) and H_2_O_2_ solely to the outer water phase, so that any 4MU reaching that compartment would be oxidized to a nonfluorescent product [45]. The emulsions containing HCD in the internal and external water phase and HRP in the external water phase showed the lowest cross-talk (Figure 3) and we therefore replicated these conditions for further experiments.

### 2.2. Sorting of Reference and Mutant ChiA Gene Libraries

Using the optimized system containing HCD in the inner phase, and HCD, HRP, and H_2_O_2_ in the outer phase, we prepared reference libraries containing 5% ChiA-positive cells mixed with 95% nonexpressing cells and compartmentalized them in double emulsions. After a reaction time of 1 h, we sorted 1000 positive events onto agar plates. However, we recovered fewer than 300 colonies from the plates and none of them were positive. Importantly, we also plated cells before sorting as a control, and despite the initial mix containing 5% positive cells, less than 1% of cells recovered from the presorting plate were positive. This suggested that the ChiA is toxic to the cells, which has previously been reported and attributed to the hydrolysis of host cell wall polysaccharides [46].

**Figure 4:**
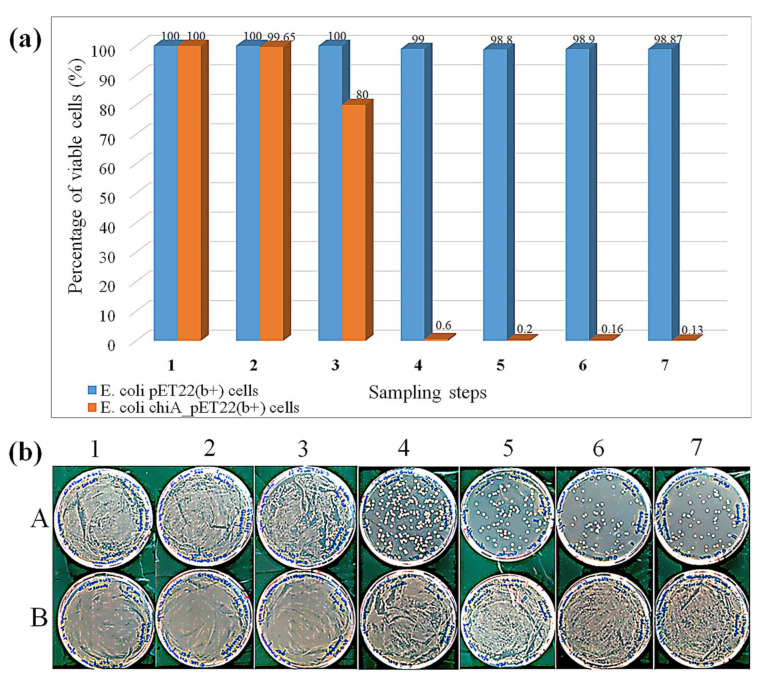
Cell viability assay at different phases of expression, cell washing, and enzymatic reaction. (**a**) Percentage of cell viability. (**b**) Plate assay, corresponding to the quantitative data shown in (a), comparing *E. coli* BL21 (DE3) cells transformed with *chi*A_pET22b or the empty pET22b(+) vector. Sampling steps on *x*-axis: 1 = 2 h expression, 2 = 4 h expression, 3 = 6 h expression, 4 = 18 h expression, 5 = 18 h expression and washing, 6 = 18 h expression, washing, and enzymatic reaction (30 min), 7 = 18 h expression, washing, and enzymatic reaction (4 h). Data are means ± standard errors representing n = 3 experiments.

Viability tests revealed that the expression of ChiA was bactericidal to *E. coli* cells after 18 h, resulting in the recovery of less than 0.6% of the colonies (Figure 4). The washing steps and the reaction with 4MUTC caused additional weak bactericidal effects. In contrast, empty vector control cells grew vigorously, as did cells expressing ChiA for 2–6 h, confirming that the main bactericidal factor was the long-term expression of ChiA. *E. coli* is already used for the production of recombinant chitinase and the product accumulates in the cytoplasm, but even in these cases some chitinase was detected in the extracellular environment, suggesting a certain degree of cell lysis [47,48,49]. These publications focused on overall expression levels or screening in agar plate or MTP assays, where a population of >10^8^ identical clones was assayed per well or spot, therefore cell lysis was not a concern. In contrast, we are testing individual cells and the maintenance of cell viability is therefore much more important. In our system, the *chiA* gene is cloned in frame with the pelB sequence such that ChiA accumulates in the periplasm, bringing it into direct contact with the cell wall and thus triggering cell lysis. This issue is probably compounded by encapsulating the cells in picoliter compartments, which results in a much higher effective chitinase concentration around the cell. We therefore reduced the *chiA* expression time to 4 h, which does not affect cell viability but still allows the detection of ChiA activity. Using these optimizations, we recreated the emulsion using the mutant library. After sorting the positive events, we achieved a 17-fold enrichment of the positive cell population.

### 2.3. Screening of Mutants Using the MTP-Based Fluorescence Assay

**Figure 5:**
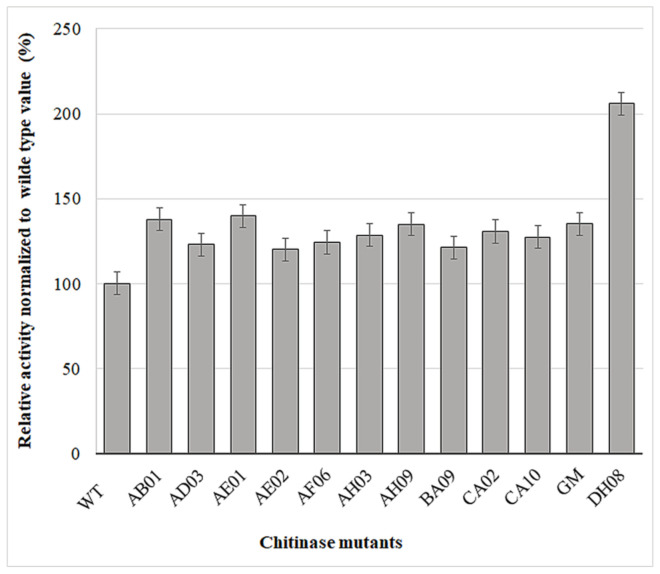
Relative activity of 12 ChiA mutants identified by FACS-based screening, normalized to wild-type ChiA. Data are means ± standard errors representing n = 3 experiments.

The mutants were initially tested using an agar plate assay. Clones showing promising activity were collected and reanalyzed using the MTP format for precise quantitation (Figure 5). We identified 12 mutants with higher activity than wild-type ChiA, one of which (DH08) was 100% more active. The positions of the mutations in all 12 clones were identified by DNA sequencing (Table 1).

### 2.4. Homology Modeling of ChiA

**Figure 6:**
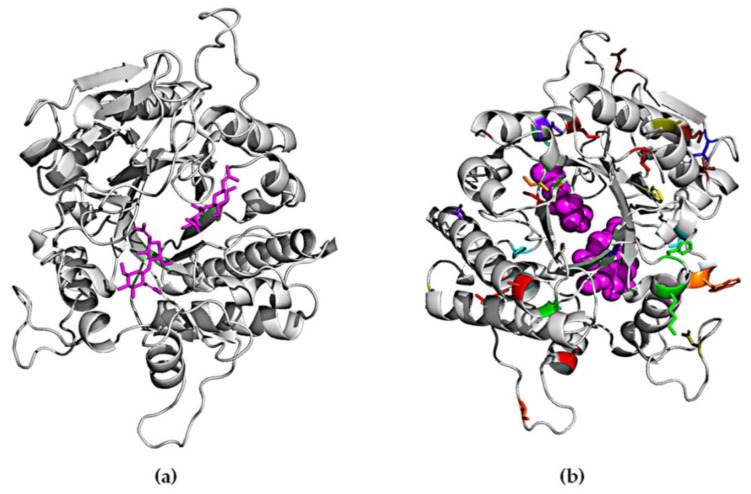
Backbone diagrams of chitinase. (**a**) Chitin substrate highlighted in the active site. (**b**) Mutations are represented by different colors corresponding to those in Table 1.

A crystal structure for ChiA is not yet available. We therefore constructed a homology model for the enzyme based on the 1edq sequence, which has 30% similarity to ChiA. Part of the N-terminal and C-terminal regions could not be modeled due to their low homology to any existing structures. Therefore, the mutations in those regions are not represented. As shown in Figure 6, the mutations obtained in this study are positioned at a distance of more than 5 Å from the active site, as evaluated using Chimera software [50], therefore we assume they do not have a direct impact on substrate binding or catalytic activity. The 12 variants we identified feature a total of 49 mutations. Only one mutation (E389D) was found in two different mutants (AB01 and GM). The rest of the mutations were unique and distributed over the entire sequence of the protein. We did not observe any clustering or any regions with a higher mutational load. Interestingly, all the mutations we recovered from a screen of 10^6^ random variants were located in loop regions or at the edges of α-helices or β-sheets, suggesting that secondary structures are highly conserved and do not tolerate substitutions.

Fourteen of the mutations we recovered (V46I, N53S, D110N, K128E, H130N, D153E, I252V, T295S, V296L, E389D, D427N, N468D, V484I, and R574Q) could be defined as conservative [51]. However, such mutations can still help to improve protein activity in the same manner as natural mutations that confer incremental increases in fitness and contribute specifically to stability [52]. The rest of the mutations were not conserved. An earlier study involving the directed evolution of chitinases, starting from the same *B. licheniformis* ChiA sequence used in our study [20], generated a variant containing five mutations, two of which were also recovered in our screen (Q369 and N468). This is interesting because the mutants in both cases were selected from a completely random library, suggesting these positions may be important for ChiA activity. Three of our mutants (P190, P436T, and S441P) involved exchanges that either removed or introduced proline residues, thus conferring the potential to change the flexibility of the polypeptide backbone. Mutations that replace charged with uncharged residues or vice versa may have a subtle effect on protein structure due to long-range electrostatic interactions [53]. We identified 16 such substitutions: K16I, K24N, W42R, D110N, K128E, H130N, D220Y, R147L, N192K, G303D, V304D, D427N, R448C, N469D, G516R, and R574Q.

All our variants contained multiple mutations, making it difficult to speculate on the importance of individual residues because there are no obvious explanations for the observed effects, as is often the case for mutants generated by directed evolution [54,55]. In order to better understand the structure–activity relationship, further investigations are required involving direct empirical testing and computational analysis. The evaluation of single mutants is currently underway, combined with molecular dynamics simulations.

## 3. Materials and Methods

### 3.1. Chemicals and Enzymes

Chemicals were purchased from Carl Roth (Karlsruhe, Germany) or Sigma-Aldrich (Taufkirchen, Germany) if not otherwise stated. NucleoSpin DNA purification kits were supplied by Macherey-Nagel (Düren, Germany). *Pfu* HF DNA polymerase, *Taq* DNA polymerase, and the GeneMorph II Random Mutagenesis kit were obtained from Agilent Technologies (Santa Clara, CA, USA). Phusion High-Fidelity DNA Polymerase and *Dpn* I were obtained from New England Biolabs (Ipswich, MA, USA).

### 3.2. Directed Evolution of ChiA and Library Preparation

The synthetic *B. licheniformis* DSM8785 *chiA* gene (GenBank accession number FJ465148) [4] was synthesized by GenScript Biotech (Piscataway, NJ, USA) and inserted into vector pET22b(+) for error-prone PCR as previously described [24].

### 3.3. MTP Assay for ChiA Activity

*E. coli* BL21 (DE3) OverExpress precultures carrying pET22b(+) or *chi*A_pET22b(+) were inoculated into lysogeny broth (LB) medium containing 0.1 mg/mL ampicillin at OD_600_ = 0.1 and incubated for 2 h at 37 °C, shaking at 160 rpm, until the OD_600_ reached 0.8–1.0. Enzyme expression was initiated by adding 1 mM isopropyl β-d-1-thiogalactopyranoside (IPTG) and incubating for 18 h as above. Cells were collected by centrifugation at 3000× *g* for 20 min, then washed four times in 0.1 M sodium acetate buffer (pH 6.0) with intervening centrifugation steps at 11,000× *g* for 1 min. Finally, the cells were resuspended in 100 µL 0.1 M sodium acetate buffer (pH 6.0) and 25 µL of the suspension was transferred to the MTP and mixed with 25 µL 0.5 mg/mL 4MUTC. The reactions were monitored on a plate reader (excitation 355 nm, emission 465 nm) with shaking at every 30 s.

### 3.4. Cell Viability Assay

Aliquots of cells (1 µL) were removed 2, 4, 6, and 18 h after the addition of IPTG and diluted with water to an OD_600_ of 0.6–0.8. Then, 1 µL aliquots from the diluted suspension were plated onto LB agar plates supplemented with 0.1 mg/mL ampicillin and incubated for 24 h at 37 °C before counting the colonies. The remaining culture after 18 h was washed by four rounds of centrifugation and resuspension in 0.1 M sodium acetate (pH 6.0) as above before resuspending in 100 µL of the same buffer to provide a nonexposed stock suspension. Next, 1 µL of the nonexposed stock was diluted to OD_600_ = 0.6–0.8, and 25 µL of this diluted suspension was mixed with 25 µL 0.5 mg/mL 4MUTC. After 30 min or 4 h, the postexposure suspension was spread on LB agar plates supplemented with ampicillin as above, to test cell viability in the presence of 4MUTC. Another 1 µL aliquot of the nonexposed stock was diluted to OD_600_ = 0.6–0.8 and spread on the LB agar plus ampicillin plates as a nonexposed control.

### 3.5. FACS Assay

The in vitro compartmentalization of bacteria was carried out as previously described [56] with the following modifications. The washed cells together with the reaction components from the MTP assay described above (25 μL) were added to 250 μL of the ice-cold oil phase comprising 1.5% (*v*/*v*) ABIL EM 90 (Evonik, Essen, Germany) in light mineral oil. The two phases were homogenized on ice in a 2 mL round-bottom cryotube for 3 min at 7000 rpm using a MICCRA D1 homogenizer (ART Prozess & Labortechnik, Müllheim, Germany). The second water phase (500 μL) containing 1.5% (*w*/*v*) carboxymethylcellulose (CMC) and 1% (*v*/*v*) Triton X-100 in PBS was added to the primary emulsion and homogenized on ice for 3 min at 10,000 rpm. We then diluted 10 µL of each emulsion in 2 mL PBS for analysis on a BD FACS DiVa flow cytometer (BD, Franklin Lakes, NJ, USA) with the following parameters: one drop enrich sorting mode, P2 gate, photomultiplier tube (PMT) 15, 355–460 nm, PMT 3-SSC (side scatter). Events sorted at a throughput of 1000 were collected on LB agar plates supplemented with 0.1 mg/mL ampicillin and incubated overnight at 37 °C.

### 3.6. Testing of Cyclodextrins and Different Oils

For initial evaluation, single emulsions were prepared from (a) 2.9% (*v*/*v*) ABIL EM 90 and 1% (*v*/*v*) Triton X-100 in PBS; (b) 2.9% (*v*/*v*) ABIL EM 90, 1% (*v*/*v*) Triton X-100, and 1.5% (*w*/*v*) CMC in PBS, or (c) 2.9% (*v*/*v*) ABIL EM 90, 1% (*v*/*v*) Triton X-100, 1% (*v*/*v*) commercial Picosurf in HEFE 7300, and 1% (*w*/*v*) SDS. In each case, the emulsion was divided into three proportions, one of which was mixed with 1 mM 4MU, another with 1 mM 4MU + 100 mM MCD, and the third with 1 mM 4MU + 100 mM HCD.

For library screening, the initial emulsion obtained by mixing 500 µL 2.9% (*v*/*v*) ABIL EM in mineral oil with 20 µL cells and 10 µL 1.32 mM 4MUTC was divided in two portions of 250 µL, one of which was mixed with 10 µL 100 mM MCD and the other with 10 µL 100 mM HCD. Both mixtures were homogenized for 3 min at 10,800 rpm before mixing with 500 µL 1% (*v*/*v*) Triton X-100 in PBS and homogenizing again as above. The resulting emulsions were each divided into three portions of 250 µL. The first portion was used without further modification (MCD or HCD in the inner water phase only). The second portion was supplemented with 10 µL 100 mM MCD or HCD in the external water phase. The third portion was supplemented with 10 µL 100 mM MCD or HCD in the external water phase as well as 50 µL 50 U/mL HRP and 25 µL 10 mM H_2_O_2_. All emulsions were diluted in PBS and analyzed by FACS, as described above.

### 3.7. Sorting and Selection of ChiA Mutants

The library emulsions were sorted after incubating them with the substrate for 1 h at room temperature. One thousand positive events were sorted and transferred to agar plates to evaluate the number of droplets containing viable cells. After 24 h, 50–100 viable colonies were transferred to fresh agar plates containing 0.5% (*w*/*v*) colloidal chitin [37] and 1 mM IPTG at 37 °C. After *chiA* expression for 48 h, the proportion of cells producing ChiA was evaluated by observing halo formation around the colonies, allowing us to calculate the overall percentage viability and chitinase expression.

### 3.8. MTP Fluorescence Assay

Three hundred colonies representing positive mutants, 20 wild-type positive controls, and 20 vector-only negative controls were transferred to 96-well MTPs containing 200 µL LB medium supplemented with 0.1 mg/mL ampicillin. The plates were incubated overnight at 37 °C, shaking at 900 rpm. Precultures (5 µL) were transferred to 200 µL LB medium containing antibiotics and incubated overnight as above. From these precultures, 20 µL of the suspension was transferred to fresh MTPs containing 200 µL LB medium supplemented with 0.1 mg/mL ampicillin. After 3 h, 200 µL of LB medium containing 0.1 mg/mL ampicillin and 2 mM IPTG were added to induce *chiA* expression. After 18 h, preliminary analysis of the mutant enzyme activity based on fluorescence allowed us to calculate the relative activity of the improved mutants compared to wild-type ChiA. The positions of the mutations were confirmed by DNA sequencing [57].

### 3.9. Homology Modeling

ChiA was modeled using SWISS-MODEL Workspace [58] and structure 1edq.1 as the template. This is a ChiA protein from *Serratia marcescens* with 31.57% sequences similarity to *B. licheniformis* DSM8785 ChiA.

## 4. Conclusions

A series of ChiA mutants with improved activity were generated using a combination of error-prone PCR for molecular evolution and flow cytometry for high-throughput screening of the resulting library. 4MUTC was selected as the fluorescent substrate for screening. The fluorescent product is hydrophobic, so we looked at multiple ways to prevent the leakage of the product from the emulsions. The best results with the lowest crosstalk were achieved using HDC, which was found to retain 4MU most efficiently. This approach was preferable to the use of a fluorogenic substrate that generates a hydrophilic fluorescent product because the latter would require a much more complex process of chemical synthesis. Our strategy will be useful for other screening applications using emulsions. It also extends the range of substrates that are compatible with high-throughput screening based on FACS.

An additional issue we encountered was the toxicity of ChiA activity in the *E. coli* host cells, which made it difficult to recover viable cells after sorting. We addressed this challenge by fine tuning the expression levels of the *chiA* gene. This approach worked well for the wild-type ChiA, but the mutants recovered from the library never exceeded the wild-type activity by more than twofold, suggesting that toxicity remains an issue for enzymes with much higher levels of activity in this assay. We are considering several strategies to overcome this drawback, including the testing of even shorter induction periods and the use of cell-free in vitro coupled transcription–translation systems which would not be dependent on cell survival. Despite these limitations, the current version of the assay enabled the recovery of 12 improved ChiA variants, in most cases featuring mutations in surface loops far from the active site. These data suggest that the activity of ChiA can be increased by introducing structural changes that have a broader effect, such as improving protein folding and flexibility (which facilitates the hydrolysis of insoluble and crystalline substrates) as well as overall stability. This is the first report describing an ultrahigh-throughput screening system for improving chitinase activity, and provides a new strategy that can lead us a step closer to finding chitinase variants suitable for the environmentally friendly degradation of chitinaceous waste in a sustainable way.

## Figures and Tables

**Table 1:** Positions of mutations in the 12 ChiA mutants identified by FACS-based screening. The sequences were analyzed using Vector NTI software. The color codes correspond to those in Figure 6.

No	Mutants	Mutation Points Number	Amino Acids Replaced (Mutations)
1	ChiA (wild-type)	-	-
2	AB01	5	D154E, F315L, E389D, S530P, G568S
3	AE01	8	V46I, P190A, N192K, T295S, N446I, G516R, T537M, R574Q
4	AE02	6	K16I, A17V, W42R, I87N, I252V, V474L
5	AD03	2	R448C, A471E
6	AH03	2	V484I, A506V
8	AH09	7	K24N, S79L, R147L, A218V, S230C, I292F, S441P
7	AF06	1	**Y362S**
9	BA09	4	L7S, N53S, V296L, V304D
10	CA02	4	D110N, P436T, A443V, N469D
11	CA10	2	**A165T, D427N**
12	GM	5	M3R, G303D, Q369L, E389D, T419M
13	DH08	3	**K128E, H130N, D220Y**

## Data Availability

Not applicable.

## Supplementary Materials

The following are available online at https://www.mdpi.com/1422-0067/22/6/3041/s1, Figure S1: Chemical structures of chitin and chitosan, Figure S2. Chemical structures of the cyclodextrins used to prevent leakage of the fluorescent product 4MU, Figure S3. FACS response of 4MU, in the presence and absence of MCD/HCD, using a perfluorinated oil (PicoSurf) and SDS detergent, in double water-oil-water emulsions.
