# Supplementary material for: A High-Throughput Screening System Based on Fluorescence-Activated Cell Sorting for the Directed Evolution of Chitinase A
Source: Int J Mol Sci. 2021 Mar 16;22(6):3041. doi: 10.3390/ijms22063041 (PMC8002391; doi:10.3390/ijms22063041)

Figure S1. Chemical structures of chitin and chitosan. (**a**) Chitin fragment comprising six *N*-acetyl-D-glucosamine residues. (**b**) Partially-deacetylated chitin (chitosan), here presented as six alternating *N*-acetyl-D-glucosamine and D-glucosamine residues. (**c**) Fully-deacetylated chitosan (poly-D-glucosamine) comprising six D-glucosamine residues. The arrows show the positions cleaved by ChiA, an endochitinase that cleaves the glycosidic bond randomly at internal sites in a chitin or chitosan polymer, downstream of an *N*-acetyl-D-glucosamine unit. The requirement for an *N*-acetyl-D-glucosamine unit means that ChiA cannot hydrolyze fully-deacetylated chitosan.

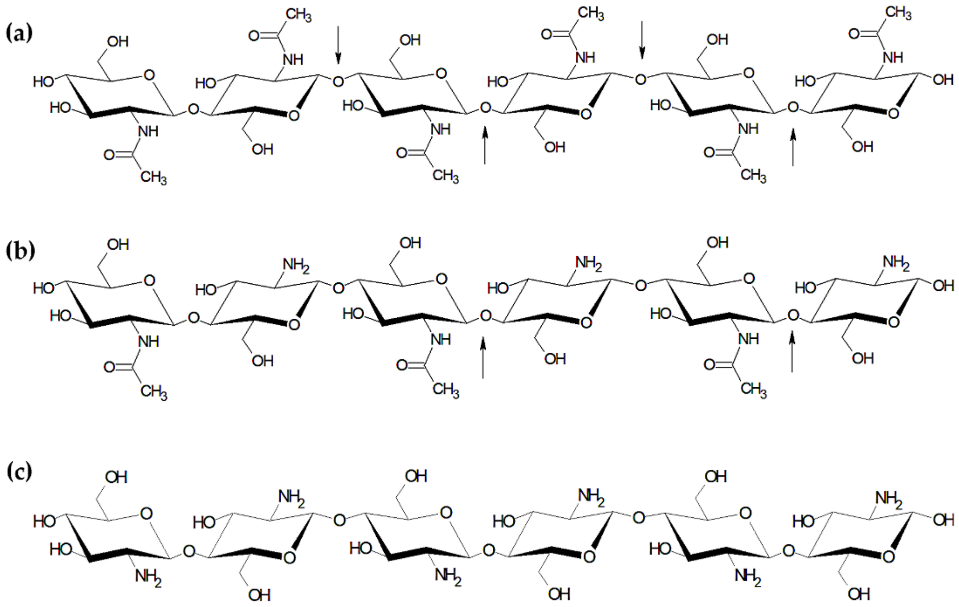

Figure S2. Chemical structures of the cyclodextrins used to prevent leakage of the fluorescent product 4MU. **(a)** Methyl-β-cyclodextrin (MCD). **(b)** 2-Hydroxypropyl-β-cyclodextrin (HCD). **(c)** Possible structure of methyl-β-cyclodextrin. **(d)** Possible structure of 2-hydroxypropyl-β-cyclodextrin.

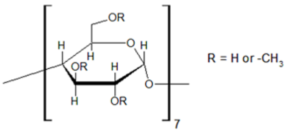

(a)

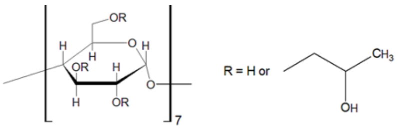

(b)

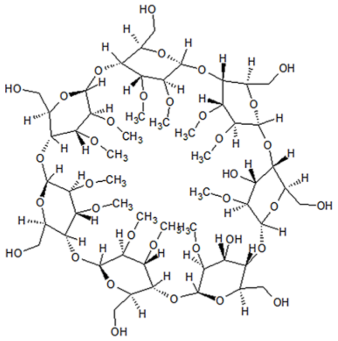

(c)

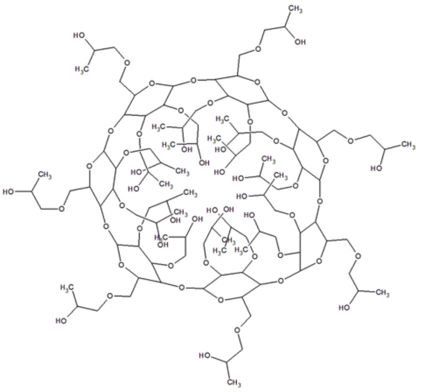

(d)

Figure S3. FACS response of 4MU, in the presence and absence of MCD/HCD, using a perfluorinated oil (PicoSurf) and SDS detergent, in double water-oil-water emulsions.

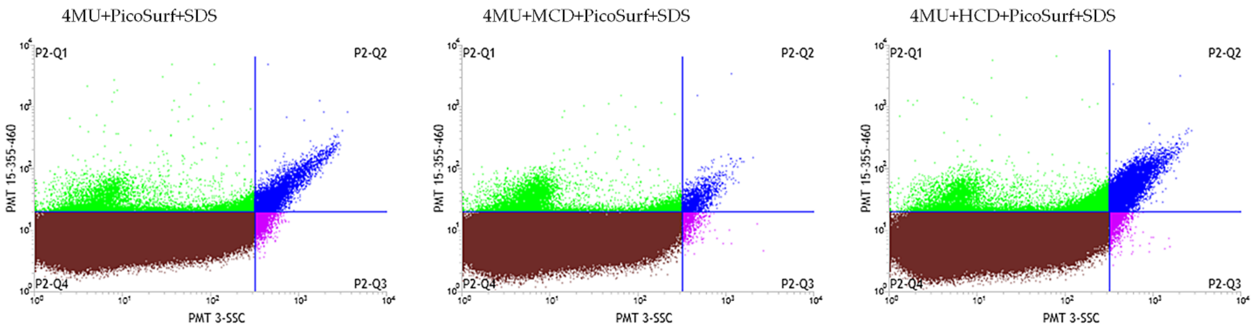

Supplement: Supplementary file 1 [file ijms-22-03041-s001.pdf]
